# (2,2′-Bipyridine-κ^2^ *N*,*N*′)bis­(4-chloro­benzoato-κ*O*)zinc

**DOI:** 10.1107/S160053681200699X

**Published:** 2012-02-24

**Authors:** Bi-Song Zhang, Hong-Line Zhu, Jun Li, Dong-Dong Dai, Yi-Bao Peng

**Affiliations:** a College of Materials Science and Chemical Engineering, Jinhua College of Profession and Technology, Jinhua, Zhejiang 321017, People’s Republic of China; b State Key Laboratory Base of Novel Functional Materials and Preparation, Science Center of Applied Solid State Chemistry Research, Ningbo University, Ningbo, Zhejiang 315211, People’s Republic of China

## Abstract

In the title compound, [Zn(C_7_H_4_ClO_2_)_2_(C_10_H_8_N_2_)], the Zn^II^ atom is coordinated by two O atoms from two 4-chloro­benzoate ligands and two N atoms from a chelating 2,2′-bipyridine (bpy) mol­ecule in a distorted N_2_O_2_ tetra­hedral geometry. The Zn^II^ atom is located on a twofold rotation axis, which also passes through the mid-point of the central C—C bond of the bpy ligand. In the crystal, weak C—H⋯O hydrogen bonds and π–π stacking inter­actions between the pyridine rings of the bpy ligands [centroid–centroid distance = 3.642 (3) Å] link the complex mol­ecules into a two-dimensional supra­molecular structure parallel to (100). An intra­molecular C—H⋯O hydrogen bond is also observed.

## Related literature

For zinc(II) complexes with substituted benzoate ligands, see: Aghabozorg *et al.* (2005 ▶); Chen *et al.* (2006 ▶); Hökelek *et al.* (2008 ▶); Lemoine *et al.* (2004 ▶); Liu *et al.* (1998 ▶); Wei *et al.* (2002 ▶, 2004 ▶); Xu *et al.* (2004 ▶); Ye & Zhang (2010 ▶); Zhang *et al.* (2009 ▶, 2010 ▶); Zhou *et al.* (2005 ▶).
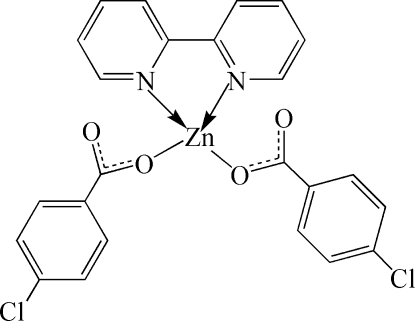


## Experimental

### 

#### Crystal data

[Zn(C_7_H_4_ClO_2_)_2_(C_10_H_8_N_2_)]
*M* *_r_* = 532.68
Monoclinic, 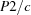

*a* = 11.453 (2) Å
*b* = 8.4896 (17) Å
*c* = 12.337 (3) Å
β = 107.12 (3)°
*V* = 1146.4 (5) Å^3^
*Z* = 2
Mo *K*α radiation
μ = 1.34 mm^−1^
*T* = 290 K
0.32 × 0.25 × 0.18 mm

#### Data collection

Rigaku R-AXIS RAPID diffractometer
Absorption correction: multi-scan (*ABSCOR*; Higashi, 1995 ▶) *T*_min_ = 0.675, *T*_max_ = 0.787
8641 measured reflections
2012 independent reflections
1560 reflections with *I* > 2σ(*I*)
*R*_int_ = 0.058

#### Refinement

*R*[*F* ^2^ > 2σ(*F* ^2^)] = 0.041
*wR*(*F* ^2^) = 0.121
*S* = 1.17
2012 reflections
150 parameters
48 restraints
H-atom parameters constrained
Δρ_max_ = 0.53 e Å^−3^
Δρ_min_ = −0.36 e Å^−3^

### 

Data collection: *RAPID-AUTO* (Rigaku, 1998 ▶); cell refinement: *RAPID-AUTO*; data reduction: *CrystalStructure* (Rigaku/MSC, 2002 ▶); program(s) used to solve structure: *SHELXS97* (Sheldrick, 2008 ▶); program(s) used to refine structure: *SHELXL97* (Sheldrick, 2008 ▶); molecular graphics: *XP* in *SHELXTL* (Sheldrick, 2008 ▶) and *DIAMOND* (Brandenburg, 1999 ▶); software used to prepare material for publication: *SHELXTL*.

## Supplementary Material

Crystal structure: contains datablock(s) I, global. DOI: 10.1107/S160053681200699X/hy2516sup1.cif

Structure factors: contains datablock(s) I. DOI: 10.1107/S160053681200699X/hy2516Isup2.hkl

Additional supplementary materials:  crystallographic information; 3D view; checkCIF report

## Figures and Tables

**Table 1:** Hydrogen-bond geometry (Å, °)

*D*—H⋯*A*	*D*—H	H⋯*A*	*D*⋯*A*	*D*—H⋯*A*
C1—H1⋯O1^i^	0.93	2.55	3.172 (6)	125
C3—H3⋯O2^ii^	0.93	2.52	3.278 (5)	139
C11—H11⋯O1^iii^	0.93	2.57	3.330 (5)	139

Symmetry codes: (i) 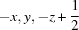; (ii) 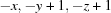; (iii) 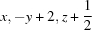.

**Fig. 1.**
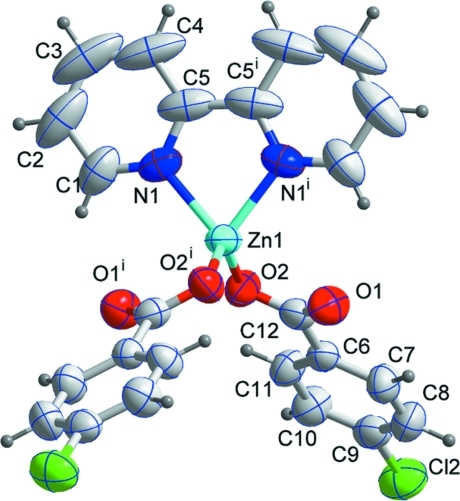
The molecular structure of the title compound. Displacement ellipsoids are drawn at the 50% probability level. [Symmetry code: (i) -x, y, -z+1/2.]

**Fig. 2.**
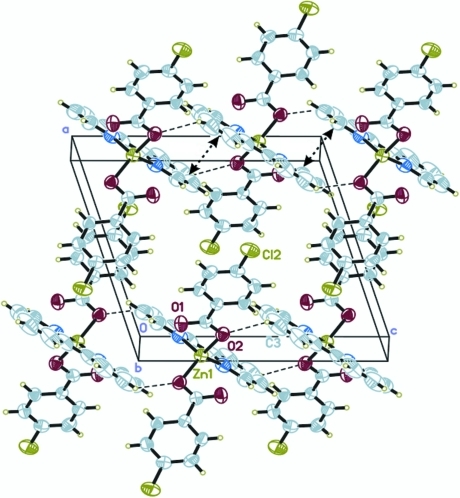
A view of the crystal packing, showing π–π interactions (dashed double arrows), with a centroid–centroid distance of 3.642 (3) Å, and C—H···O hydrogen bonds (dashed lines).

## supplementary crystallographic information

### Comment

Transition metal complexes with biochemical molecules show interesting physical and/or chemical properties, which may find applications in biological systems. The structure–function–coordination relationships of arylcarboxylates in Zn^II^ complexes of benzoic acid derivatives may alos be changed depending on the nature and positions of the substituted groups on the benzene ring. Zinc(II) complexes with substituted benzoic acid ligands have been reported (Aghabozorg *et al.*, 2005; Chen *et al.*, 2006; Hökelek *et al.*, 2008; Lemoine *et al.*, 2004; Liu *et al.*, 1998; Wei *et al.*, 2002, 2004; Xu *et al.*, 2004; Ye & Zhang, 2010; Zhang *et al.*, 2009, 2010). In this paper, we report the synthesis and structure of the title complex.

In the title compound, the Zn^II^ atom is coordinated by two O atoms and two N atoms from two 4-chlorobenzoate ligands and one 2,2'-bipyridine (bpy) molecule in a distorted ZnN_2_O_2_ tetrahedral geometry. The O1 atom of the 4-chlorobenzoate ligand has a weak interaction with the Zn^II^ atom [Zn1···O1 = 2.602 (3) Å]. A similar distance has been observed [Zn1···O2 = 2.653 (7)Å] (Zhou *et al.*, 2005). The Zn^II^ atom is located on a twofold rotation axis, which also passes through the mid-point of the C5—C5^i^ bond [symmetry code: (i) -x, y, -z+1/2] of the bpy ligand. The bpy ligand exhibits nearly perfect coplanarity (r.m.s. deviation = 0.049 Å). In the crystal, weak C—H···O hydrogen bonds (Table 1) and π–π stacking interactions [centroid–centroid distance = 3.642 (3) Å] between adjacent bpy ligands link the complex molecules into a two-dimensional supramolecular structure parallel to (100).

### Experimental

ZnCl_2_ (0.0687 g, 0.504 mmol) was dissolved in appropriate amount of water and then 1M Na_2_CO_3_ solution was added. ZnCO_3_ was obtained by filtration, which was then washed with distilled water for 5 times. The freshly prepared ZnCO_3_, 2,2'-bipyridine (0.0388 g, 0.273 mmol) and 4-chlorobenzoic acid (0.0396 g, 0.255 mmol), CH_3_OH/H_2_O (v/v = 1:2, 15 ml) were mixed and stirred for 2 h. The resulting cream suspension was heated in a 23 ml Teflon-lined stainless steel autoclave at 433 K for 97 h. After the autoclave was cooled to room temperature within 43 h, the solid was filtered off. The resulting filtrate was allowed to stand at room temperature and slow evaporation for 6 weeks afforded colorless block single crystals.

### Refinement

H atoms were placed in calculated positions and refined as riding atoms, with C—H = 0.93 Å and *U*_iso_(H) = 1.2*U*_eq_(C).

### Crystal data

**Table:**

[Zn(C_7_H_4_ClO_2_)_2_(C_10_H_8_N_2_)]	*F*(000) = 540
*M**_r_* = 532.68	*D*_x_ = 1.543 Mg m^−^^3^
Monoclinic, *P*2/*c*	Mo *K*α radiation, λ = 0.71073 Å
Hall symbol: -P 2yc	Cell parameters from 6683 reflections
*a* = 11.453 (2) Å	θ = 3.0–25.0°
*b* = 8.4896 (17) Å	µ = 1.34 mm^−^^1^
*c* = 12.337 (3) Å	*T* = 290 K
β = 107.12 (3)°	Block, colorless
*V* = 1146.4 (5) Å^3^	0.32 × 0.25 × 0.18 mm
*Z* = 2	

### Data collection

**Table:**

Rigaku R-AXIS RAPID diffractometer	2012 independent reflections
Radiation source: rotation anode	1560 reflections with *I* > 2σ(*I*)
Graphite monochromator	*R*_int_ = 0.058
ω scans	θ_max_ = 25.0°, θ_min_ = 3.0°
Absorption correction: multi-scan (*ABSCOR*; Higashi, 1995)	*h* = −13→13
*T*_min_ = 0.675, *T*_max_ = 0.787	*k* = −9→10
8641 measured reflections	*l* = −14→14

### Refinement

**Table:**

Refinement on *F*^2^	Primary atom site location: structure-invariant direct methods
Least-squares matrix: full	Secondary atom site location: difference Fourier map
*R*[*F*^2^ > 2σ(*F*^2^)] = 0.041	Hydrogen site location: inferred from neighbouring sites
*wR*(*F*^2^) = 0.121	H-atom parameters constrained
*S* = 1.17	*w* = 1/[σ^2^(*F*_o_^2^) + (0.051*P*)^2^ + 0.432*P*] where *P* = (*F*_o_^2^ + 2*F*_c_^2^)/3
2012 reflections	(Δ/σ)_max_ < 0.001
150 parameters	Δρ_max_ = 0.53 e Å^−^^3^
48 restraints	Δρ_min_ = −0.36 e Å^−^^3^

### Special details

**Table:**

Geometry. All esds (except the esd in the dihedral angle between two l.s. planes) are estimated using the full covariance matrix. The cell esds are taken into account individually in the estimation of esds in distances, angles and torsion angles; correlations between esds in cell parameters are only used when they are defined by crystal symmetry. An approximate (isotropic) treatment of cell esds is used for estimating esds involving l.s. planes.
Refinement. Refinement of F^2^ against ALL reflections. The weighted R-factor wR and goodness of fit S are based on F^2^, conventional R-factors R are based on F, with F set to zero for negative F^2^. The threshold expression of F^2^ > 2sigma(F^2^) is used only for calculating R-factors(gt) etc. and is not relevant to the choice of reflections for refinement. R-factors based on F^2^ are statistically about twice as large as those based on F, and R- factors based on ALL data will be even larger.

### Fractional atomic coordinates and isotropic or equivalent isotropic displacement parameters (Å^2^)

**Table:**

	*x*	*y*	*z*	*U*_iso_*/*U*_eq_	
Zn1	0.0000	0.78102 (6)	0.2500	0.0564 (2)	
Cl2	0.59680 (10)	1.35611 (14)	0.61254 (11)	0.0912 (4)	
O1	0.1788 (3)	0.9459 (3)	0.2186 (2)	0.0738 (7)	
O2	0.1206 (2)	0.8948 (3)	0.3696 (2)	0.0650 (7)	
C1	−0.1402 (4)	0.6021 (6)	0.3867 (4)	0.0889 (13)	
H1	−0.1636	0.7013	0.4048	0.107*	
C2	−0.1780 (5)	0.4710 (8)	0.4330 (5)	0.122 (2)	
H2	−0.2269	0.4814	0.4808	0.146*	
C3	−0.1427 (7)	0.3267 (9)	0.4076 (6)	0.144 (3)	
H3	−0.1657	0.2369	0.4394	0.172*	
C4	−0.0731 (6)	0.3138 (6)	0.3351 (5)	0.121 (2)	
H4	−0.0493	0.2150	0.3168	0.145*	
C5	−0.0377 (4)	0.4489 (4)	0.2888 (3)	0.0827 (13)	
N1	−0.0713 (3)	0.5916 (3)	0.3171 (3)	0.0686 (8)	
C6	0.2915 (3)	1.0622 (4)	0.3941 (3)	0.0512 (8)	
C7	0.3756 (4)	1.1328 (5)	0.3489 (3)	0.0679 (10)	
H7	0.3691	1.1184	0.2726	0.082*	
C8	0.4688 (4)	1.2242 (5)	0.4152 (4)	0.0741 (11)	
H8	0.5246	1.2720	0.3842	0.089*	
C9	0.4779 (3)	1.2436 (4)	0.5275 (4)	0.0629 (10)	
C10	0.3952 (4)	1.1763 (5)	0.5739 (3)	0.0690 (10)	
H10	0.4017	1.1915	0.6501	0.083*	
C11	0.3019 (3)	1.0853 (4)	0.5063 (3)	0.0614 (9)	
H11	0.2455	1.0391	0.5375	0.074*	
C12	0.1909 (3)	0.9613 (4)	0.3207 (3)	0.0553 (8)	

### Atomic displacement parameters (Å^2^)

**Table:**

	*U*^11^	*U*^22^	*U*^33^	*U*^12^	*U*^13^	*U*^23^
Zn1	0.0659 (4)	0.0427 (3)	0.0618 (4)	0.000	0.0206 (3)	0.000
Cl2	0.0755 (7)	0.0805 (7)	0.1037 (9)	−0.0151 (6)	0.0048 (6)	−0.0030 (6)
O1	0.097 (2)	0.0728 (16)	0.0544 (16)	−0.0092 (15)	0.0258 (14)	−0.0083 (13)
O2	0.0722 (16)	0.0637 (15)	0.0639 (16)	−0.0123 (13)	0.0275 (13)	−0.0042 (12)
C1	0.080 (3)	0.105 (3)	0.081 (3)	−0.019 (2)	0.021 (2)	0.025 (2)
C2	0.104 (4)	0.151 (4)	0.097 (4)	−0.054 (4)	0.009 (3)	0.050 (4)
C3	0.165 (6)	0.116 (4)	0.110 (5)	−0.073 (4)	−0.021 (4)	0.058 (4)
C4	0.161 (5)	0.059 (3)	0.103 (4)	−0.040 (3)	−0.023 (3)	0.024 (3)
C5	0.104 (3)	0.0480 (18)	0.069 (3)	−0.013 (2)	−0.018 (2)	0.0074 (17)
N1	0.073 (2)	0.0545 (17)	0.070 (2)	−0.0075 (15)	0.0080 (16)	0.0108 (14)
C6	0.0590 (19)	0.0428 (16)	0.055 (2)	0.0036 (15)	0.0216 (15)	0.0031 (14)
C7	0.077 (3)	0.072 (2)	0.064 (2)	−0.006 (2)	0.035 (2)	−0.0049 (19)
C8	0.070 (3)	0.075 (3)	0.086 (3)	−0.012 (2)	0.038 (2)	0.002 (2)
C9	0.061 (2)	0.0511 (19)	0.074 (3)	0.0037 (17)	0.0154 (19)	0.0019 (17)
C10	0.079 (3)	0.072 (2)	0.054 (2)	−0.007 (2)	0.0172 (19)	−0.0008 (18)
C11	0.068 (2)	0.064 (2)	0.056 (2)	−0.0094 (18)	0.0234 (17)	0.0027 (17)
C12	0.065 (2)	0.0435 (17)	0.058 (2)	0.0065 (16)	0.0180 (17)	−0.0001 (15)

### Geometric parameters (Å, º)

**Table:**

Zn1—O2	1.954 (2)	C4—H4	0.9300
Zn1—N1	2.082 (3)	C5—N1	1.347 (5)
Zn1—O1	2.602 (3)	C5—C5^i^	1.466 (10)
Cl2—C9	1.740 (4)	C6—C11	1.368 (5)
O1—C12	1.233 (4)	C6—C7	1.384 (5)
O2—C12	1.272 (4)	C6—C12	1.506 (5)
C1—N1	1.329 (6)	C7—C8	1.377 (6)
C1—C2	1.377 (6)	C7—H7	0.9300
C1—H1	0.9300	C8—C9	1.368 (6)
C2—C3	1.356 (10)	C8—H8	0.9300
C2—H2	0.9300	C9—C10	1.368 (5)
C3—C4	1.366 (11)	C10—C11	1.382 (5)
C3—H3	0.9300	C10—H10	0.9300
C4—C5	1.394 (6)	C11—H11	0.9300
			
O2—Zn1—O2^i^	120.76 (15)	C2—C3—C4	119.6 (5)
O2—Zn1—N1	110.75 (11)	C2—C3—H3	120.2
O2^i^—Zn1—N1	114.15 (11)	C4—C3—H3	120.2
O2—Zn1—N1^i^	114.15 (11)	C3—C4—C5	119.9 (6)
O2^i^—Zn1—N1^i^	110.75 (11)	C3—C4—H4	120.1
N1—Zn1—N1^i^	78.9 (2)	C5—C4—H4	120.1
O2—Zn1—C12	28.13 (10)	N1—C5—C4	119.6 (5)
O2^i^—Zn1—C12	107.47 (11)	N1—C5—C5^i^	115.9 (2)
N1—Zn1—C12	135.29 (12)	C4—C5—C5^i^	124.5 (4)
N1^i^—Zn1—C12	101.44 (12)	C1—N1—C5	119.8 (4)
O2—Zn1—C12^i^	107.47 (11)	C1—N1—Zn1	125.5 (3)
O2^i^—Zn1—C12^i^	28.13 (10)	C5—N1—Zn1	114.6 (3)
N1—Zn1—C12^i^	101.44 (12)	C11—C6—C7	118.8 (3)
N1^i^—Zn1—C12^i^	135.29 (12)	C11—C6—C12	120.9 (3)
C12—Zn1—C12^i^	107.90 (14)	C7—C6—C12	120.3 (3)
O2—Zn1—O1	55.55 (9)	C8—C7—C6	120.9 (4)
O2^i^—Zn1—O1	91.94 (10)	C8—C7—H7	119.6
N1—Zn1—O1	153.21 (11)	C6—C7—H7	119.6
N1^i^—Zn1—O1	86.47 (11)	C9—C8—C7	119.0 (4)
C12—Zn1—O1	27.42 (9)	C9—C8—H8	120.5
C12^i^—Zn1—O1	104.74 (10)	C7—C8—H8	120.5
O2—Zn1—O1^i^	91.94 (10)	C10—C9—C8	121.2 (4)
O2^i^—Zn1—O1^i^	55.55 (9)	C10—C9—Cl2	119.5 (3)
N1—Zn1—O1^i^	86.47 (11)	C8—C9—Cl2	119.3 (3)
N1^i^—Zn1—O1^i^	153.21 (11)	C9—C10—C11	119.3 (4)
C12—Zn1—O1^i^	104.74 (10)	C9—C10—H10	120.4
C12^i^—Zn1—O1^i^	27.42 (9)	C11—C10—H10	120.4
O1—Zn1—O1^i^	114.90 (12)	C6—C11—C10	120.8 (4)
C12—O1—Zn1	76.2 (2)	C6—C11—H11	119.6
C12—O2—Zn1	105.5 (2)	C10—C11—H11	119.6
N1—C1—C2	122.1 (5)	O1—C12—O2	122.8 (3)
N1—C1—H1	118.9	O1—C12—C6	120.7 (3)
C2—C1—H1	118.9	O2—C12—C6	116.5 (3)
C3—C2—C1	118.9 (6)	O1—C12—Zn1	76.4 (2)
C3—C2—H2	120.5	O2—C12—Zn1	46.41 (16)
C1—C2—H2	120.5	C6—C12—Zn1	162.9 (3)

Symmetry code: (i) −*x*, *y*, −*z*+1/2.

### Hydrogen-bond geometry (Å, º)

**Table:**

*D*—H···*A*	*D*—H	H···*A*	*D*···*A*	*D*—H···*A*
C1—H1···O1^i^	0.93	2.55	3.172 (6)	125
C3—H3···O2^ii^	0.93	2.52	3.278 (5)	139
C11—H11···O1^iii^	0.93	2.57	3.330 (5)	139

Symmetry codes: (i) −*x*, *y*, −*z*+1/2; (ii) −*x*, −*y*+1, −*z*+1; (iii) *x*, −*y*+2, *z*+1/2.
